# “The problem is ours, it is not CRAIDS’ ”. Evaluating sustainability of Community Based Organisations for HIV/AIDS in a rural district in Zambia

**DOI:** 10.1186/1744-8603-8-40

**Published:** 2012-11-28

**Authors:** Aisling Walsh, Chishimba Mulambia, Ruairi Brugha, Johanna Hanefeld

**Affiliations:** 1 Department of Epidemiology and Public Health Medicine, Division of Population Health Sciences, Royal College of Surgeons in Ireland, Beaux Lane House, Lower Mercer Street, Dublin 2, Ireland; 2 Institute of Economic and Social Research, University of Zambia, Lusaka, Zambia; 3 Department of Global Health and Development, Faculty of Public Health and Policy, London School of Hygiene and Tropical Medicine, London, UK; 4 School of Social and Political Science, University of Edinburgh, Edinburgh, UK

**Keywords:** Community Based Organisations, HIV/AIDS, Sustainability, World Bank, Zambia, Community participation, Care and support services

## Abstract

**Background:**

While sustainability of health programmes has been the subject of empirical studies, there is little evidence specifically on the sustainability of Community Based Organisations (CBOs) for HIV/AIDS. Debates around optimal approaches in community health have centred on utilitarian versus empowerment approaches. This paper, using the World Bank Multi-Country AIDS Program (MAP) in Zambia as a case study, seeks to evaluate whether or not this global programme contributed to the sustainability of CBOs working in the area of HIV/AIDS in Zambia. Lessons for optimising sustainability of CBOs in lower income countries are drawn.

**Methods:**

In-depth interviews with representatives of all CBOs that received CRAIDS funding (n = 18) and district stakeholders (n= 10) in Mumbwa rural district in Zambia, in 2010; and national stakeholders (n=6) in 2011.

**Results:**

*Funding*: All eighteen CBOs in Mumbwa that received MAP funding between 2003 and 2008 had existed prior to receiving MAP grants, some from as early as 1992. This was contrary to national level perceptions that CBOs were established to access funds rather than from the needs of communities. Funding opportunities for CBOs in Mumbwa in 2010 were scarce.

*Health services*: While all CBOs were functioning in 2010, most reported reductions in service provision. Home visits had reduced due to a shortage of food to bring to people living with HIV/AIDS and scarcity of funding for transport, which reduced antiretroviral treatment adherence support and transport of patients to clinics.

*Organisational capacity and viability*: Sustainability had been promoted during MAP through funding Income Generating Activities. However, there was a lack of infrastructure and training to make these sustainable. Links between health facilities and communities improved over time, however volunteers’ skills levels had reduced.

**Conclusions:**

Whilst the World Bank espoused the *idea* of sustainability in their plans, it remained on the periphery of their Zambia strategy. Assessments of need on the ground and accurate costings for sustainable service delivery, building on existing community strengths, are needed before projects commence. This study highlights the importance of enabling and building the capacity of existing CBOs and community structures, rather than creating new mechanisms.

## Background

It has long been acknowledged that communities are at the forefront in fighting the HIV pandemic. Their strengths can lie in a deep understanding of the contexts and impacts of HIV and AIDS, community solidarity, and practical, locally appropriate and experience-based solutions [1]. In countries that reversed major HIV epidemics, such as Uganda, Senegal and Thailand, non-governmental and community-based responses have played a vital role in this success; and in many countries the community response preceded the national response [2]. Community Based Organisations (CBOs)^a^ develop services and interventions that are culturally appropriate and more responsive to the preferences and needs of communities than those provided by state sectors [3]. Initiatives to fight HIV/AIDS existed at the community level in Zambia as early as 1986 [4] and in 2008 civil society provided 30% of VCT services, 80% of treatment care and support services, and 70% of orphans and vulnerable children (OVC) services [5].

The Alma Ata declaration of 1978 acted as a starting point in recognising the importance of community participation in health, through stating *“primary health care requires and promotes maximum community and individual self-reliance and participation in the planning, organisation, operation and control of primary health care”*[6]. In the context of HIV, this was reinforced by the UN Declaration on HIV/AIDS (UNGASS) [7], the Millennium Development Goals and the World Health Report, 2008 [8]. Lawn et al. [9] state that community participation is the principle of Alma-Ata that has been most neglected over the last three decades. The sustainability of health programmes has been the subject of empirical studies over the last three decades [10], and two published studies have assessed the sustainability of HIV/AIDS services in Zambia [11,12]. However, neither focuses on the sustainability of CBOs for HIV, which is an area where more evidence is needed, given their major input in this area [13,14].

This paper, using the World Bank Multi-Country AIDS Program (MAP) in Zambia as a case study, seeks to evaluate whether or not this global programme contributed to the sustainability of CBOs working in the area of HIV in Zambia. It makes recommendations for promoting the sustainability of community based services for HIV care and support. Harman [15] posits that the World Bank has remained beyond the realm of scrutiny, as it has positioned itself as a ‘benevolent donor’. Her paper argues that lip service is often paid to sustainability, without adequate consideration and assessment of what actually needs to be sustained. Assessments of need on the ground and accurate costings for sustainability of service delivery are needed. We conclude with recommendations on appropriate funding mechanisms for CBOs and, while based on a case study from one rural district in Zambia, the depth of the analysis means that the findings have wider relevance for CBOs in low and middle income countries. A set of principles, rather than a single model or formula is proposed for ensuring the sustainability of CBO HIV programmes, as each community must base sustainability plans on the needs, contexts and experiences of its’ individuals and families.

### Frameworks for community based services/health interventions

Debates around optimal approaches in community-based health interventions have centred around the dichotomy of utilitarian versus empowerment approaches [16,17]. Within a utilitarian or target oriented framework, community participation is viewed as a means to the end of health improvements. A utilitarian approach and evaluation of a community intervention or programme seeks to quantify the changes observed in the health status of the population, or in the performance indicators that best reflect the objectives of the programme. In this approach, the community is the object of the intervention [17].

The empowerment framework sees participation as an objective or end in itself. Empowerment interventions or programmes aim to enable communities to gain access to and control of health care resources, through promoting their capacity to mobilise and grow as a community [18]. Within the empowerment framework, indicators of success of the intervention lie not so much (or not only) in the coverage and impact of the services provided by CBOs, but in the effects on and responses of the community and the increase in its ‘social capital’ [14,19]. Many definitions of social capital exist [20]. Putnam [19] describes it as *‘features of social organisation such as networks, norms and social trust that facilitate coordination and cooperation for mutual benefit.’*

Three criteria distinguish the target-oriented from the empowerment frameworks: i) who makes the decisions on resource allocation (professional versus community members); ii) the desired outcome, or importance attached to the outcome (health status versus social change); and iii) the methods used in outcome assessment (quantitative versus qualitative) [17]. In empowerment approaches, the community is the subject rather than the object of social change. Those who espouse the empowerment framework have been criticised for making unrealistic assumptions about the abilities of the poor and marginalised to effect change [21] whilst ignoring the wider social and political circumstances which make empowerment unrealistic [22].

### Definitions and debates around sustainability

‘Sustainability’ as a concept entered development discourse, especially in papers on primary health care and the health sector, from the early 1990s [23-26]. A systematic review of empirical research on health sustainability, between 1980 and 2008, reported 84 studies of which 24 were from developing countries [10]. Definitions include both programmatic and financial components of sustainability, and the term has multiple, sometimes contested meanings [10], which can be divided into those focusing on maintaining: a) health benefits/programmes (utilitarian) and b) community capacity (empowerment). They also identify factors affecting sustainability such as project description and implementation, attributes of the organisational setting and factors in the environment [10]. *“For donors, it may mean that project costs can be borne by locals without further international aid; for policymakers it may mean that the initiative in question has to be continually reinvented and reinvigorated to stand the test of time.”*[16]*.* We have chosen that by Sarriot et al. [27] as the most encompassing definition: 

*“A contribution to the development of conditions enabling individuals, communities and local organisations to express their potential, improve local functionality, develop mutual relationships of support and accountability, and decrease dependency on insecure resources (financial, human, technical, informational) in order for local stakeholders to negotiate their respective roles in the pursuit of health and development, beyond a project intervention”*[27]*.*

Traditional project approaches to sustainability consider sustainability during or even at the end of the implementation process [27]. Whatever the definition, the literature shows a positive relationship between community participation and sustainability [17,28,29]. Ooms et al. [30] have argued *“that the global charity model, to help people help themselves temporarily rather than to create a sustained pool of redistributed funds, has provided an insufficient response to the challenges preventing MDG progress”.* Only a small number of studies have explored and attempted to identify the factors determining the sustainability of CBOs. Gruen et al. [10] suggest that the determinants of sustainability need to be explored and *“the interactions between drivers and programme components in a particular context.”*

### HIV/AIDS in Zambia

Zambia is a lower middle income country in Southern Africa which, in 2010 had a population of 13,047,000. The most recently available figures show an adult HIV/AIDS prevalence rate of 14.3% n 2007 [31]. The death rate from HIV/AIDS amongst the adult population has decreased by 66.7% between 2002 and 2011, and 90% of adults in need of antiretroviral treatment (ART) were accessing it. However only 28% of children needing ART were actually receiving it [31].

Zambia is heavily dependent on donor funding for implementing the vast majority of its HIV/AIDS services, although domestic budget allocations to health have increased from 20% of the total budget in 2004 to 48% in 2009 [32]. In 2006, 62% of external funding to HIV/AIDS control in Zambia was provided by the US President’s Emergency Plan for AIDS Relief (PEPFAR) and 13% by the Global Fund to Fight AIDS, TB and Malaria [33]. The minimum budget required to finance adequate levels of health care in poor countries has been estimated by the Commission on Macroeconomics and Health to be US$35 per person per year [34]. Between 1995 and 2002, Zambian Government health spending averaged $12 per person, per year and although had increased significantly in dollar terms in 2006, this reflected a significant appreciation of the exchange rate of the Zambian Kwacha [35].

### Background to World Bank MAP in Zambia

The World Bank Multi-County AIDS Program in Africa (MAP), which was launched in 2000, was the first Global Health Initiative that was established to fight HIV and AIDS, and was followed by the Global Fund in 2002 and PEPFAR in 2004. MAP marked a shift from traditional World Bank lending, in that it was designed to be community oriented, demand-driven and multi-sectoral. Projects were intended to fit within the recipient country’s development strategy and the Bank’s strategy for lending in that country [15]. Its funding ethos aimed to recognise HIV related community activities already underway by providing financial support to existing projects, and seed money for new forms of community-led development [15].

A pre-condition to qualify for MAP funding was that the recipient country would make a commitment to disburse 40-60% of MAP funds to Civil Society Organisations (CSOs). In 2002, the World Bank provided the Zambian Government with a grant of US $ 42 million to support the National HIV/AIDS Strategic Plan 2001–2005 through the MAP-funded Zambia National Response to HIV/AIDS (ZANARA) project, which was to run between 2003 and 2008. One component of ZANARA was the Community Response to AIDS (CRAIDS), a funding mechanism that provided resources for community based HIV and AIDS programmes and accounted for 35% of total MAP commitments in Zambia.

CRAIDS also supported the district HIV and AIDS response through funding the District AIDS Task Force (DATF) and a District AIDS Coordination Advisor (DACA) for all districts. Provincial and District AIDS structures had been established prior to the commencement of the ZANARA project. An important feature of ZANARA was that CRAIDS would operate within the existing system and not set up additional structures. In 2003, the World Bank funded 22% of the national response to HIV and AIDS control. By 2005 this represented only 9% of the response, as support from the Global Fund had arrived. It represented 1% of the national response at the time of ZANARA’s close in 2008, when PEPFAR had become the major funder of HIV and AIDS control activities in Zambia [36]. The World Bank’s Africa Region HIV/AIDS Agenda for Action*,* 2007*–*2011 [37], resulted in a change of Bank strategy and in 2008 the Zambian Government was offered a loan of US $20 million as a follow-on to the earlier grant, as Zambia no longer qualified for a grant due to the completion of the Heavily Indebted Poor Country Initiative. The Government rejected the loan offer and World Bank MAP funding to HIV ceased in August 2008.

### Frameworks for assessing sustainability

Several frameworks for assessing sustainability in Community Health Initiatives were explored as a basis for this study [24,38-40]. The framework which is used in this paper to assess the sustainability of CBOs for HIV and AIDS is an adaptation of the Child Survival Sustainability Assessment Framework [27]. Other frameworks were deemed unsuitable, as they did not include health services as a component. Sarriot et al’s framework, which is used to organise the findings in this paper, consists of three dimensions and six components of sustainability (see Table 1), to which we added a fourth dimension, funding. The second component of Dimension Four (ecological, human, political, and policy environment) is covered in the introduction and discussion, as there were limited findings under these themes and they are beyond the scope of this paper.

**Table 1:** **Framework for assessment of sustainability of Community Based Organisations (adapted from Sarriot et al., 2004**[27]

	***Dimension***	***Component***	***Explanatory notes***
***1***	***Funding***	*D1.* Concerned with having sufficient funding to continue programme objectives. It follows the trajectory of funding pre-programme to programme funding to post programme funding outcomes	Sarriot et al’s framework [27] incorporated funding into the organisational dimension, specifically organisational viability. However we consider that it warrants separate consideration, considering the centrality of financing to programme sustainability
***2***	***Health and social services***	*D2.1 Health and social services approach:* availability, cost, accessibility and appropriateness of services.	Health services are defined in this study in the broadest terms, as CBOs for HIV and AIDS provide social services in addition to health services, such as nutrition and education support. Sarriot et al’s framework also includes quality and coverage. Within this study it was not possible to report on coverage as this is not incorporated into planning and M&E for care and support services in Zambia. This is discussed in the paper. Assessment of service quality was deemed beyond the scope of the study
		D2.2 *Health outcomes:* represents the improvement of the health of the population	
***3***	***Organisational***	*D3.1 Organisational capacity:* the capacity that is needed to exist within local organisations to maintain local services and activities.	
		*D3.2 Organisational viability:* the capacity of an organisation for continuing effectiveness, in particular organisational dependency/interdependency and interconnectedness.	
***4***	***Community and social ecological conditions***	D4.1 Community competence/capacity: overlapping elements that affect the community such as social cohesion and collective efficacy – ‘community competence’	‘Community competence’ is defined as ‘a range of functions of community life (leadership, communication skills, conflict management, sense of community, internal participation) which contribute to the competence of the community.’ [41]
		D4.2 Ecological, human, economic, political and policy environment: national and regional economic and political policies, and ecological conditions	The authors consider that this is most appropriately dealt with in introduction and discussion

## Methods

### Study design

A case study was deemed the most appropriate approach for an in-depth exploration of the sustainability of CBOs delivering HIV and AIDS care and support services. Case study research is typically useful to answer the ‘how’ or ‘why’ questions about relationships between individuals and between communities, and to reflect changes of events over time. It allows the researcher to explore individuals, organisations and communities, and interventions ranging from simple to complex [42]. According to Gerring [43], case studies may be more useful than cross-case studies when a subject is being encountered for the first time. In this case this is the first known empirical analysis of sustainability of CBOs for HIV/AIDS in Zambia. A case should be defined by: (a) *time and place –* in this study it was Mumbwa rural district, in the pre-CRAIDS era, the CRAIDS era and post-CRAIDS era; b) *time and activity –* the various dimensions of the Framework, see Table 1 and Results section, and; c) *definition and context* – this case study focused on CBOs in Mumbwa that had been funded by MAP [44].

### Study setting

Mumbwa rural district was selected as a district where CRAIDS had funded many CBOs, and where the District AIDS Task Force (DATF) remained active, despite CRAIDS cessation. Mumbwa is a rural district located in Central Province, with a population of 218,328 [45]. There were other funders of HIV and AIDS care and support activities present in Mumbwa district during the period of interest, 2007–10. Selecting one district allowed for a more in-depth exploratory study of the issues than would have been possible with structured surveys across several districts. The limitation is that the findings may not be representative of the post-CRAIDS experiences of HIV and AIDS support CBOs across other districts of Zambia.

### Data collection and analysis

All CBOs in Mumbwa district that had received CRAIDS funding were identified with the support of the District AIDS Coordination Advisor (DACA) and representatives from all of these were interviewed by the authors (n = 18). District level interviewees included representatives of the District AIDS Task Force (DATF), Mumbwa District Council and Mumbwa District Commission, and representatives from the Community AIDS Task Forces (n =10). Interviews were conducted with key national stakeholders including senior representatives of government ministries and agencies, and of bilateral and multilateral development agencies (n = 6). Interviews were recorded and transcribed. A thematic analysis was conducted, on which these findings are based. A draft of the report summary and recommendations was tabled at a dissemination meeting in Lusaka in March 2011 where senior representatives of the National AIDS Council (NAC), the relevant Ministries and many of the major donors and development agencies attended and discussed the findings. Ethical clearance for the study was granted by the University of Zambia.

## Results

### Dimension One – funding

#### CRAIDS funding

Most CBOs that had been approved for funding received the CRAIDS funds between 2005 and 2007. However, some CBOs reported that there had been delays in the receipt of funds – sometimes up to three years. Some CBOs received a lump sum while others received their funds in instalments, usually over one or two years. The size of grants per organisation ranged from 38 million Zambian Kwacha (approximately US $ 8,000) to 72 million Zambian Kwacha (approximately US $15,000), which they received in payment vouchers or cheques. Although the initial target was to fund 350 projects, CRAIDS funded 1,800 community initiatives that were selected from 5000 applications, country-wide.

All eighteen CBOs in Mumbwa that received CRAIDS funding between 2003 and 2008 had existed prior to receiving CRAIDS grants, some from as early as 1992. Some of these CBOs were initiated in communities as women’s clubs to respond to the emerging HIV epidemic. Of those that received CRAIDS funding, nine had no external funding prior to CRAIDS. Previously, they had relied for funds primarily on animal rearing, farming and other income generating activities (IGAs) such as knitting, sewing and cooking, as well as through their own membership donations and fundraising. The reality on the ground contrasted with the perception of some national level respondents, which was that CBOs emerged in order to access CRAIDS funds, without proper plans in place to serve the community of people and families living with HIV and AIDS. CBO respondents reported that these organisations, which were all in place before CRAIDS funding was established, had been formed due to an increase of HIV and AIDS in their communities and in particular due to the rise of orphans and vulnerable children (OVCs).

Other funders of CBOs in Mumbwa included international NGOs, organisations funded through the Global Fund, and the Government’s Constituency Development Fund, which funded small grants and income generation activities. Whilst this Fund was still active at the time of data collection, it was reported by the CBOs that the other donors were no longer funding services in Mumbwa in 2010. When first launched, CRAIDS funding opportunities were made known to CBOs primarily through the DACA and the CRAIDS Regional Facilitator^b^. In general the CRAIDS project was viewed positively by the CBOs. CRAIDS did not impose the conditions that other sources of funding required, such as audited accounts; the absence of this pre-condition was reported to have made funding more accessible to CBOs. The principle requirement was that organisations register with the district and open a bank account.

#### CRAIDS cessation

A report commissioned by the National AIDS Council (NAC) in 2007 advised the World Bank and Government to plan an exit strategy and recommended that CRAIDS should move to NAC to be a specialised unit that would serve as the link between government and civil society [3]. Many CBO representatives (n=11) reported that the DACA had informed them that CRAIDS funding was coming to an end, although he himself had received no formal notification of this. There had also been a general awareness amongst most CBOs from the start that CRAIDS funding was available for a limited time period only. Most CBOs were clear from the outset about the total amount that they were being awarded and the duration of their grants; and many received a certificate of grant completion. However, some CBOs said they were given no notice and that CRAIDS ceased abruptly, leaving them with no time to put alternative plans in place, as they had been told that they would be eligible to apply for another round of funding. They reported uncertainty about whether or not funding would continue. *“Once CRAIDS had closed, communities were left on their own and there was a period of disorientation and confusion amongst the communities.” (DATF representative)*

#### The current funding gap

There was consensus among interviewees that the funding opportunities for CBOs in Mumbwa in 2010 were scarce and had decreased since the end of CRAIDS in 2008. Most CBOs were not aware of other sources of funding that they could apply for. In 2010, there was little knowledge of PEPFAR amongst CBOs in Mumbwa, or of the different grants that PEPFAR provided which CBOs would be eligible to apply for. A number of CBOs voiced a perception that the Zambia National AIDS Network (ZNAN), the civil society umbrella body funded by the Global Fund which was funding CBOs to provide HIV and AIDS services, made it difficult for communities to access funds.

National level respondents in late 2010 spoke of other national funding schemes, which might or could fill the gap left by CRAIDS. For example, the Ministry of Community Development, Mother and Child Health takes guidance from NAC on HIV and runs cross-cutting programmes such as a food security programme, and a women and development programme. The Ministry of Education runs a public welfare assistance scheme, which includes the provision of school fees and uniforms, and a school feeding programme. However, none of these schemes were mentioned by any of the CBO representatives we interviewed in Mumbwa.

The social cash transfer scheme was another avenue through which some people living with HIV and OVCs were reported by national level respondents to be benefiting directly from; the scheme was being rolled out slowly throughout the country and had not yet reached Mumbwa at the time of the study. One national level respondent put this forward as an alternative to funding CBOs, whereas others outlined the importance of maintaining support for the community in addition to the cash transfer scheme.

### Dimension Two - health and social services

### D2.1 Health and social services approach

Field appraisal by the District AIDS Task Force (DATF)^c^ was used as the method of assessing how well applications from CBOs for CRAIDS funding support would meet the needs on the ground. CRAIDS laid out target specifications for grant applications. For example, the minimum number of OVCs to be targeted to be eligible for CRAIDS funding to support a community school was 100; and the minimum number of individuals in a specific target vulnerable group to attract funding was 20 [46]. The importance of having a local leader with in-depth knowledge of the community and the ability to estimate and if possible quantify community need was mentioned by many of the CBOs as being the key to the community response. 

*“Because DATFs are based locally, they did desk and field appraisals and were able to identify areas of weakness which went beyond the check lists of the tools for monitoring.”* (National level respondent)

Services provided by CBOs in Mumbwa included prevention, care and support to those in need, specifically by providing home-based care (HBC), nutrition support, general OVC support, peer education and HIV counselling (see Table 2). Whilst some CBOs specialised in one support service type, for example home-based care or OVC support, many provided multiple services. Transport (mainly bicycles) for promoting antiretroviral treatment (ART) adherence, travel to the hospital/clinic and for care givers was also widely provided by CBOs. Prevention services focused primarily on sensitisation and education of communities about HIV and AIDS, of which CBOs were the most widespread providers. International NGOS such as World Vision and Child Fund also delivered HIV care and support services, but with defined catchment areas that did not cover entire districts, which was often the case with CRAIDS.

**Table 2:** Services provided by Community Based Organisations

**Home-based care:** ARV adherence, nutritional support, counselling, cleaning and washing, delivering food	n=13
**Sensitisation:** HIV prevention, stigma/discrimination reduction including youth drama groups and awareness campaigns	n = 6
**OVC support:** subsidies for nutrition support (primarily school feeding programmes), school uniforms, school fees, counselling	n = 12

CRAIDS programme staff and the DATF specified which services were to be provided and the income generating activities that could be funded through CRAIDS. Many of the CBOs were unhappy with these conditions and not all received funding for the services for which they applied. For example, one CBO applied for funds to purchase cattle, but was given funding to engage in poultry farming. In some cases, support to IGAs did not meet the demands of the local market, for example some CBOs were provided with funding for a hammer mill, where there were already several operating in the area, making it hard for the CBO to find customers. In another case, a CBO that applied for funds to do home-based care was funded to undertake awareness campaigns, as mapping exercises undertaken by the DATF had revealed that there were sufficient numbers of CBOs providing HBC in that area.

Some respondents praised the DATF for preventing duplication of services in Mumbwa. District and national level respondents attributed the requirements on CBOs to change the focus of their planned activities to the need to prevent duplication of services in the district and to ensure that there was a proper distribution and availability of services across the district. Many respondents thought that CRAIDS should have channelled more funds into sustainable plans. However, interviewees used the term sustainability broadly, without specifying what they meant by it.

While all CBOs were still functioning at the time of community interviews in mid 2010, all except one reported reductions in service provision. The biggest obstacle, reported by all CBOs, was the lack of transport – for HBC givers, for ART adherence support, for HIV counselling, and to bring patients to the hospital/clinics. While CRAIDS funding had provided bicycles, many of these were now beyond repair, resulting in some care-givers walking for up to 10 kilometres to reach their clients. Some reported that they had discontinued visits to homes of people living with AIDS as they no longer had food to bring to houses. Examples of a scaling-down in services included a reduction of OVC support from 100 to 70 orphans and from 300 to 110 care givers. CBOs described the difficult decisions they were forced to make around which OVCs to support, and which to neglect - one organisation chose children who had lost both parents to AIDS; another chose the first 50 children on their list out of 700, neglecting those who had been identified more recently. Out of 12 organisations providing HBC, only one reported that their service levels had remained unchanged since they stopped receiving CRAIDS funding.

One area where outcomes were measured by the CBOs themselves was in school attendance and nutrition. A large decrease in nutritional support for schools was reported, and had been cut completely in two schools. Also, CBOs were no longer able to buy uniforms for children, which were a pre-requisite for attending school. This had resulted in children dropping out of school.

*“For the vulnerable and orphans there is a change because when CRAIDS was funding us there was a feeding programme. Right now we are failing to feed the children. We are also finding it very difficult to keep or manage 100 children. Some of them have stopped coming to school. We are struggling to keep the school going and to retain the number of pupils attending class. ”* (CBO representative)

District interviewees described the focus of HIV/AIDS service in Mumbwa as shifting more towards treatment and prevention to the neglect of care and support services since CRAIDS had ceased. Some perceived that the priority within Mumbwa district in 2010 was to ensure people received ART, with little or no emphasis on nutrition and support to the community as a whole, nor to the families affected by HIV and AIDS.

### D2.2 Health outcomes

Health outcomes directly attributed to these support services are more difficult to measure and have not been routinely measured in Zambia as part of CBOs' activities. The National AIDS Reporting Forms (NARFs) and Ministry of Health reporting systems (Health Management Information Systems) do not capture health outcomes. NARFs however capture service utilisation indicators such as numbers of OVCs receiving care and support from CBOs, and numbers of individuals provided with HIV palliative care. In many cases, CBOs reported numbers of services provided by CBOs and numbers of volunteers, without referring to actual reports (see D2.1 above). However two CBOs showed summaries of monthly totals of services provided which were charted with precision on their walls. No CBO showed evidence of planning or estimating numbers of clients receiving services according to catchment areas or coverage rates. The CRAIDS Implementation Manual [46] did not mention catchment areas or outcomes, except to state that *“methods of analysis might include for example a comparison before and after the project”,* which suggests inadequate attention to measuring outcomes at the design stage.

### Dimension three- organisational

### D3.1 Organisational capacity

One of the significant benefits of CRAIDS mentioned by interviewees at all levels lay in its training of volunteers in service provision, business management, and how to run Income Generation Activities (IGAs). This was reported by most community respondents to have increased the capacity of CBOs and enabled community empowerment. All CBOs funded by CRAIDS reported capacity-building activities over the CRAIDS funding period. On the business side, capacity was built in management skills, banking issues and reporting. Training took place primarily in the form of workshops.

*“The capacity that was built for me was how to do things in the bank. I had never seen how it looks inside the bank. I did not know what a cheque looked like and I never had the knowledge of why cheques are rejected. I even knew how to withdraw and bank the money.”* (CBO representative)

CBO representatives also reported building one another’s capacity by passing on skills from training initially received through CRAIDS. This was reported to have promoted sustainability post CRAIDS and boosted morale amongst volunteers. The ZANARA project had specified that the change in capacity of communities to manage their own development will be monitored [46]. This was not mentioned by either CBOs or district representatives.

#### Volunteers – the pulse of the community response

Volunteers were and had continued to be at the heart of the care and support services provided by CBOs for people living with and affected by HIV and AIDS. Without them, CBOs would not be able to deliver or sustain service provision. They have provided a wide range of services including psycho-social counselling, HBC, treatment adherence support, peer education and sensitisation. The numbers of volunteers in individual CBOs in Mumbwa ranged from 10 to 110. Volunteers described their motivation as coming from a desire to improve their community through determination and hard work. They would walk long distances to provide HBC to families and to bring reports to the DATF. Some reported volunteering with the same organisation for up to ten years. Interviewees explained that people living with HIV and AIDS preferred to be counselled by voluntary groups in their own community, which they saw as the big comparative advantage of CBOs.

*“These people come from the communities, they live with these people who have these problems… because there is that closer link with the people that are living in the community itself and that builds the confidence of people who are chronically ill, because they live with these people.”* (District representative)

While a condition of CRAIDS funding was that it could not be used to provide allowances or incentives to volunteers, some received allowances from monies raised through IGAs, though they were not formally paid.

A decrease in the morale of volunteers since CRAIDS funding ceased, was frequently lamented by interviewees, which they attributed primarily to a lack of materials to carry out their work, such as HBC kits, washing and cleaning materials. CBO respondents reported that some had resigned due to work overload. Respondents also reported that skills had been lost over time, particularly since CRAIDS ceased, as some of those who had received training had moved on to other activities or out of the district. Others reported a loss of capacity over time, due to HIV medical knowledge and guidelines becoming outdated.

### D3.2 Organisational viability

#### Effectiveness and sustainability of IGAs

According to some CBOs and district respondents, CRAIDS funding has provided some CBOs in Mumbwa with the opportunity to become sustainable, and most had engaged in IGAs to enable or increase the scale of service provision. This was the principal CRAIDS strategy for addressing sustainability. One condition of CRAIDS funding was that all proposals for vulnerable groups should have an IGA element. These activities ranged from rearing poultry, pigs and goats; as well as vegetable and crop farming, cooking, knitting and sewing.

Provision of hammer mills to mill maize, was one of the main IGAs in Mumbwa, although some CBO representatives reported that they generated little profit, due to frequent break-downs and the lack of resources to repair the mills. Animal rearing was also reported to be unsustainable due to a lack of markets within the district. Only two CBO representatives reported that the IGAs that they established had continued to flourish and had enabled them to maintain their level of HIV and AIDS support services, following cessation of their CRAIDS grants.

Some CBOs complained that they had either unsuccessfully applied for IGAs or were not permitted to spend as much as they wanted on these activities, which illustrates that CBOs were themselves thinking about and were concerned about sustainability from the time they sought CRAIDS funding. A number of respondents at all levels viewed that more money should have been allocated to IGAs and that CRAIDS’ sustainability plans were generally weak. Again others complained about the type of IGAs being prescribed by CRAIDS as being in some cases unsuitable, as discussed here above. However, there were also positive reports of successful IGAs. The purchase of a hammer mill through CRAIDS support enabled one CBO to open a grocery shop. Vegetable farming enabled CBOs to produce food to support people living with HIV and AIDS through HBC programmes for a set time period.

Physical infrastructure is generally poor in Mumbwa. Some groups did not have access to adequate nearby markets, and roads and bridges in the area were often impassable. This meant depending on the small markets within the village for selling the produce produced through IGAs. It was reported that too many IGAs were located in small geographical areas, creating too much competition amongst CBOs and commercial entities. If a community observed that a CBO was making money on a hammer mill, then they also sought to do the same without looking at whether the market was in place for this to occur.

*“Each time a community decided on an IGA and it was making returns, then competition came in from new comers. The outsiders wanted to do also exactly the same thing. So they lost on the market.”* (District stakeholder)

#### Organisational interdependency/interconnectedness

Many CBOs described positive working relationships with other CBOs, non-government organisations (NGOs), hospitals and clinics in Mumbwa. The relationship was primarily in the form of reciprocal arrangements, which established and strengthened the linkages between communities and health facilities: referring/bringing sick people from communities to hospitals for treatment, because of illnesses and/or to support ART adherence; and the hospital or clinic, in-turn, referred patients to CBOs for home-based care.

The relationship between the district hospital and CBOs in Mumbwa changed over the decade, 2000 to 2010, due to improved coordination between district and community levels. CBOs can now approach hospital staff with questions relating to the medical conditions of the people they serve, and there is an established link between the community-based counsellors and ART programmes. Some of the CBO caregivers have been given positions on hospital committees and the hospital has lists of all the care givers who work in the area. Counsellors from the CBO reported working in the clinic as a team alongside medical staff.

Some CBOs described cooperating with area associations outside of HIV, such as Womens’ Associations and World Vision Community Care Coalitions. The District AIDS Coordination Advisor (DACA) facilitated this in many instances. A number of CBOs did not cooperate with one another and displayed no knowledge of other organisations operating in their catchment area. Others cooperated once a year only, in the organisation of World AIDS Day events. Despite the current funding gap, interviewees believed that the support systems and linkages that they had established with the support of the CRAIDS funding had remained strong at the community level.

### Dimension four - community and social ecological conditions

### D4.1 Community competence/capacity

#### Importance of continuing the HIV and AIDS community response

There was a perception among national level interviewees that most CBOs no longer existed since CRAIDS had ceased. While this may be the case in some other districts, *all* CBOs that had received funding in Mumbwa between 2005 and 2008 were still in existence and functioning in 2010. One Community AIDS Task Force (CATF) representative spoke about promoting sustainability as being at the core of its strategy. They instilled the ethos that external funding, including support from the district, was additional.

*“So we are very successful because we have not believed in living on sponsorship. We have believed that we must be self sustainable in our own small way because that’s the only way we can fully exist. Because if we normally depend on sponsorship or funding outside our community then we may not function and that’s the policy we have. We told most of the support groups these are just there to help, the problem is ours, it is not CRAIDS’, it’s not for Family Trust and it’s not for the district*.” (CATF representative)

Respondents reported the importance of the community response to HIV which was praised for being the “only way to fight the pandemic” through community determination and hard work. Care givers had closer links as they lived within the community, and in essence *are* the community.

*“I think one of the strengths of the programme was that it gave people at the community level an opportunity to identify what the real issues affecting them were and at the same time it also provided people with the opportunity in finding a solution using their own methodologies of addressing whatever issues they had prioritised.”* (National level stakeholder).

## Discussion

Green [47] suggests that grants should seek to develop problem-solving skills and community leadership and confidence rather than to seek to institutionalise programmes that may become *‘sterile bureaucracies’*. This is echoed by Doyle and Patel [48] who describe it as the survival of the organisation for survival’s sake. While CRAIDS funding was project based, the funding mechanism recognised the importance of building the capacity of the organisation, through facilitating and then funding a bottom-up, community-articulated needs approach, supported by community IGAs, with CBOs being the main implementers of the projects / interventions. CRAIDS built on existing capacities by funding CBOs that had already been active in the communities.

The general trend in Zambia however, during the period of Global Initiative support to HIV programmes, has been to move towards top-down project support and away from investing in institution and community-building. Finances and materials are provided for project-based activities by donors and cooperating partners but not for overheads to contribute to institutional functioning [3]. The ZANARA project, through the CRAIDS funding mechanism, attempted to build on existing community capacity. With greater demands for CBOs to produce results and show visible impact, which comes particularly from donors, the pendulum has shifted further towards a utilitarian approach. This has not meant that participation was seen as an end in itself, either by the World Bank or by the communities themselves. CBOs were formed to improve lives of people living with HIV and their families. Most frameworks to evaluate sustainability do not consider health services as a component [24,39], instead paying more attention to organisational/community capacity. This suggests that the literature attaches more importance to the empowerment framework.

Harman [49], reporting on World Bank MAP supported programmes in Kenya, Tanzania and Uganda, proposed that under this programme, CBOs were merely the implementers of projects, thus following a more utilitarian framework. This Zambia case study suggests that the World Bank (ZANARA-CRAIDS) support succeeded in delivering on many elements of the empowerment framework by placing the community at the centre of health service delivery, with the need to build the capacity of such groups.

For primary health care, the debate of the last three decades focused on selective (or vertical) versus comprehensive (horizontal) health service delivery. Vertical health programmes are focused on a single issue or disease, where health activities occur in parallel and in addition to routine primary care activities [50]. Integrated programmes (also known as horizontal programmes) *“tackle overall health problems on a wide front and on a long term basis through the creation of a system of permanent institutions commonly known as general health services.”*[51]. Integrated health care provides care for interrelated health problems for entire populations. Recent years have seen a shift towards combining the strengths of both approaches [9].

Our interest in the vertical versus integrated debate is in how it relates to sustainability. Atun et al. [52] suggest that limited evidence does not allow for clear conclusions about when vertical approaches are desirable. Specifically in relation to sustainability, two studies [53,54] found that vertical programmes create unfavourable conditions for sustainability once donor funding ceases, and a decrease in community self-reliance. The limited evidence shows that vertical programmes are effective as a temporary time-limited measure (the key term here is temporary). While the dichotomy between vertical and horizontal is not as rigid in practice as in theory [55,56], horizontal approaches are seen as fostering more holistic approaches to health that are more aligned to local needs.

Most CBOs implementing HIV care and support activities in Mumbwa have been vertically funded, meaning that funding agencies and NGOs at a higher level selected, designed and funded the HIV activities that the CBOs undertook. A number of studies [24,26,57] have found that vertical programmes are less likely to be sustained than programmes that are well integrated with existing (community) systems and structures. Vertical programmes focus resources and activities on well-defined goals, but are less likely to attract indigenous sources of funding, making them vulnerable to demise when external funding ends [26].

Harman [49] found that organisations that had expanded their practices were able to attract funds from a wider range of sources, as donors perceived them to have a more holistic approach in responding to the epidemic. Also, there are less transaction costs for a donor who funds one group that engages in multiple activities, than if funding several organisations to undertake different activities [49]. Funding 1,800 CBO HIV-related projects represented a significant effort on the part of ZANARA-CRAIDS; and by 2011, donors in Zambia were shifting their focus from HIV to broader poverty-alleviation interventions such as social cash-transfer schemes. CBOs in Mumbwa remained within their niche focus of a specific care or support activity for HIV/AIDS, through the decade.

Integrating HIV/AIDS into other development initiatives, for example around Mother and Child Health and other priorities that are once again on the agenda of donors and the Zambian Government, could be a logical strategy for CBOs to continue to attract funding and build sustainability. Whatever the changes in focus at the national level, from Government and donors, CBOs will continue to be particularly well placed to build on existing relationships of trust with communities.

A key challenge for community participation in health lies in how it should be institutionalised with the formal health service. An integrated health system needs to incorporate a community and population dimension, which CBOs are best placed in Zambia and elsewhere in Africa to support. These are different and essential roles. The Brazilian Family Health Programme has institutionalised community health committees, as part of municipal health services to sustain local participation [58], ensuring that community participation does not become an alternative to, but an integral part of the state’s responsibility for health care delivery to the population.

An ideal world of community participation in health would be side-by-side involvement of community members with health care professionals and a responsible sharing of both power and responsibility [16]. Findings in this study showed the CRAIDS funding had supported the development of supportive links between the formal (health facilities) and informal (communities and volunteers) health sectors, supporting treatment adherence and effective healthcare seeking behaviour. This appears to have been an unintended and important effect, which may not have been captured in the World Bank’s evaluation of its MAP project in Zambia [36]. It appears that lessons from the CRAIDS era are being incorporated into Zambia’s National Community Health Worker Strategy [59], recognising the importance of the work carried out by community health workers for the effective running of the health system. However, other successful components of the CRAIDS project, such as the delivery of care and support by CBOs to people living with and affected by HIV/AIDS, have been neglected by donors following the cessation of the CRAIDS funding.

Lehman and Sanders [58] wrote that there is little evidence that volunteerism can be sustained for long periods of time. Community volunteers and health workers in lower and middle income countries expect and require an income. Community needs and demands often require full-time health workers, and volunteers need to spend time on other breadwinning activities. Evidence points to higher attrition rates associated with volunteers than with formal health workers [58].

CBOs rely on volunteerism because Government and donors are reluctant to fund costs outside of core services to a target group, and rarely support payments to community level workers [3]. The ZANARA-CRAIDS 2006 Annual Report [60] stated that trained volunteers were unwilling to participate in outreach programmes without being paid allowances. This was shown not to be the case in Mumbwa, where most volunteers worked for no allowances, except those that the CBOs had generated through IGAs. However, these allowances were themselves not sustainable, due to the unsustainability of the IGAs. Other factors are essential to sustainability, including ensuring that volunteers know that they are appreciated and are being fairly compensated for out of pocket expenses and losses of earnings. In general, volunteer labour and capital costs are locally generated resources that have a better chance of being sustained, if mobilised through community / CBO-driven responses to community problems such as HIV and AIDS.

CBO sustainability depends on the commitment of those volunteers who make the services happen. Findings from this study showed that health facility staff had begun to accept and appreciate the importance and usefulness of the CBO volunteer in providing health services. Other studies have shown that lay counsellors relieve the workload of overstretched health care workers [61]. According to CBO representatives in this study, health workers recognised and appreciated the services provided by lay counsellors. However, while commitment amongst existing volunteers was high, decreases in numbers of volunteers were reported across all CBOs.

Community participation and so-called ‘soft services’, such as those provided by CBOs in Mumbwa, are often perceived as less measurable and therefore more difficult to evaluate [9]. An internal evaluation of the World Bank [62] stated that poor performing projects had no targeting mechanism for reaching the poor and recommended that the World Bank create new incentives for monitoring and evaluation for both the Bank and the borrower. This would include requirements for baseline data, evaluation designs for pilot activities as well as evaluation of main project activities on an ongoing basis. It would be more accurate to state that the Bank evaluation did not succeed in identifying *how* communities targeted the poor and those in their midst who were most in need of support. NAC Zambia has recognised that the lack of quantified or collated M&E data from civil society means that its contribution to the national response is often underestimated and as a result it has been difficult to argue for appropriate resource allocations to the sector [63].

Deciding what should be sustained at the end of a project cycle should begin with an assessment of needs *and* existing community strengths (its social capital) before the start of the project; followed by measurement of achievements during and at the end of the project cycle. Torpey et al. [11] suggest that quality assurance tools should be based on national standards. Measurements of the costs of the activities that contributed to those achievements, and the costs of maintaining core activities needed for sustaining achievements, are also essential. There is no evidence to show that this occurred in Mumbwa, although the field appraisal approach can be considered to be an informal or proxy method of measurement. The CRAIDS implementation manual [46] states that there will be a focus on “impact indicators”, however there is no evidence to show that these were developed. This does not mean that little or nothing was achieved, or that efforts to identify successes and generate useful lessons – qualitatively if not quantitatively – are not of value. The greater inherent risk of bias in qualitative evaluations may have precluded the World Bank from identifying and ‘making more’ of such positive lessons; as may have an inherent preference for quantitative measurement and gold standard evaluation design methods.

Globally, frameworks, assessments and tools exist for carrying out sustainability analyses of programmes [10]. However, there is little evidence to show that these sustainability assessments and tools are an integral part of donors funding mechanisms. There was disagreement between CBOs and district officials in Mumbwa as to what HIV care and support services were needed. Sustainability assessments from the outset might have avoided this and could have been an important first step towards evaluating what should be sustained for CBO services in Mumbwa. Individual CBOs were not well placed to identify IGAs with a good chance of longer term success and lacked the capacity, technical advice and support to translate good ideas into sustainable IGA programmes. While the CRAIDS application procedure required that each target should include an IGA element [46], in reality only about 45% of all community projects included an IGA [36].

The difficulty in costing for service sustainability for ‘soft services’ is reflected in the lack of studies addressing the subject. A paper by USAID analyses costs from a CBO programme in Zambia for children affected by HIV/AIDS, providing a comprehensive set of services. Health and nutrition were the most costly programmatic areas and psychosocial support was one of the least costly programmatic areas [12]. There is no evidence from CRAIDS or World Bank documents that a costing of sustainability of those services took place in Zambia.

A particularly important feature of the HIV-support CBOs in Mumbwa that received funding was that they *all* pre-dated CRAIDS funding; and while their ability to provide specific HIV care related services increased with CRAIDS funding, the project was able to build on existing structures. These CBOs continued to function and deliver HIV support services, though at lesser capacity, following the cessation of CRAIDS funding. Therefore, it seems likely that this level of sustainability was due at least in part to CRAIDS having engaged with and supported pre-existing community structures, as it also worked with existing district, provincial and national level systems.

## Conclusions

This paper concludes with an evaluation of the achievements and shortfalls of the CRAIDS-supported CBO approach for combating HIV in Mumbwa, comparing these with the model and components of sustainability outlined earlier in Sarriot et al’s definition [27]:

A. *Pre-existing capacity of CBOs:* CBOs had the capacity to organise themselves and had a track record of service delivery before they started receiving CRAIDS funding. The representatives of the CBOs, other district and to some extent national key informants were of the view that the CRAIDS model improved CBO/volunteer capacity to deliver and scale up services and to organise themselves. The downside was that, when the funding ceased, there was little to show in terms of capacity to sustain this scaled-up service delivery coverage.

B. *Develop mutual relationships of support and accountability*: The DACA played an important role in linking organisations, especially in forging and supporting new linkages between communities (informal) and district (formal public sector) structures. However, many CBOs did not create horizontal connections with one another, and it is less clear to what extent the vertical linkages were a result of the CRAIDS model and the result of the pre-CRAIDS DACA and DATF model, on which CRAIDS built.

C. *A decreased dependency on insecure resources:* during the CRAIDS period, CBOs in receipt of support could focus on service delivery and were not vulnerable to reductions in support from other projects and funding sources. While all of the CBOs continued to exist and function after CRAIDS support ceased, service levels dropped for nearly all CBOs and funding insecurities had returned. As these organisations had pre-dated CRAIDS funding, it is likely that their resilience to reduced levels and loss of funding may be related to this fact and that one lesson for future funding or ‘ building of capacity’ could be the importance of building on pre existing structures, especially CBOs that have emerged from within the communities.

D. *In order for local stakeholders to negotiate their respective roles in the pursuit of health beyond a project intervention:* All the CBOs identified and surveyed preceded and remained in existence after the end of CRAIDS, which was always intended to be for only a defined period. It could be argued that these CBOs were already sustainable before receiving CRAIDS support; and the relatively short period of the CRAIDS project meant that it had not made them overly dependent on it. Catterson and Claes [64] have argued that longer periods of funding actually decrease the prospect of sustainability.

### National level sustainability – missed opportunity post CRAIDS?

Sustainability for CBOs providing HIV support services at the local level is very much dependent on what is happening at the national level. When ZANARA was launched in 2003, the CRAIDS component was embedded into the Zambia Social Investment Fund (ZAMSIF). This was carried out to enable CRAIDS to benefit from experiences gained by ZAMSIF and its predecessors, especially around community development approaches [3]. The Zambian Government then indicated its willingness to provide funding for the CRAIDS component at a total of US$1.5 million for the remainder of 2008 after project closing and approximately US$1.7 million in the 2009 budget [36] but this was not sustained into 2010. To add to this, a corruption scandal in 2009 meant that only 39% of total donor pledged amount was actually disbursed. The Zambian Government could have ensured institutional sustainability if decisive action had been taken in the immediate aftermath of the closure of ZANARA/CRAIDS. This did not occur despite the fact that the World Bank declared that ownership of the process by the Zambian Government was very high [36].

The World Banks’ own evaluation of the ZANARA/CRAIDS project [36] stated that: *“a firm decision on the institutional arrangements to sustain CRAIDS was not made. There was a fear that the enduring legacy of the CRAIDS Project may be lost including the institutional memory, experience and human resources which made CRAIDS effective and efficient”*. It may be that the relatively large levels of funding to HIV around 2008–09, through Global Fund and PEPFAR, led Government to conclude that HIV was being taken care of. Studies on community participation in Latin America in the 1980s found that it was often motivated by ideological and political factors that had little to do with improving health [16,65]. Our findings from a rural African district show a different picture in that all CBOs were in existence prior to CRAIDS funding became available and continued after the funding ceases, showing that community mobilisation wins out over “rent seekers” and briefcase NGOs [66].

### World Bank as a model for future sustainability?

The international relevance of this study lies in the question: is the CRAIDS model one that should be replicated in other countries and indeed in the future in Zambia? Whilst the World Bank espoused the *idea* of sustainability in their plans, it was more on the periphery rather than at the heart of their Zambia strategy. Empowerment of CBOs is possible but one needs to invest substantively to enable meaningful participation, as the differing levels of capacity between the different CBOS interviewed shows. Important lessons to be learned from CRAIDS include: the importance of involving community groups in sustainability plans from the outset; incorporating a sustainability assessment into needs assessments and funding allocation decisions; and the importance of providing technical advice and then supporting effective income generating activities at the community level. Even in the absence of a formal assessment of need, it is clear that with people living longer due to increased antiretroviral treatment coverage, the demand for care and support services will actually increase over time.

Study findings highlighted two mechanisms for providing future funding and support from the national level to HIV and AIDS care and support activities at the community level in Zambia. Recommendations that came from the national level respondents focused on either a) integrated or b) vertical mechanisms.

a) **The first option would be to mainstream HIV and AIDS care and support services within broader poverty alleviation funding channels,** such as existing schemes within the Ministry of Community Development, Mother and Child Health, Ministry of Education and Ministry of Health. This approach would need to establish and protect accessible mechanisms and ensure that communities, families and people living with HIV and AIDS receive the specialised support services they need within broader poverty funding mechanisms. CBOs, as an integral part of this, would need to directly access funds to provide these services, and receive the capacity supports to ensure service quality.

b) ***The second option is to retain a separate funding stream for community HIV and AIDS care and support activities***, as a continuation of – or building on the best features and lessons learned from – the CRAIDS model. Through this, poverty alleviation and support activities for those affected by and living with HIV and AIDS could be undertaken through a poverty alleviation channel (option a, which was the preferred option of some large donors), while more specialised AIDS care and support activities (counselling services, treatment adherence, HBC and others) could be supported through an AIDS support channel.

Whichever mechanism is chosen, or if the two are combined, it is certain that sustainability of CBOs will continue to require the support of donors in the medium to long term. CBOs will continue to play an important part in community support activities, even with the existence of social cash transfer schemes, which are becoming more popular, not only within Zambia, but in many developing countries [67]. However, people living with HIV and AIDS will continue to need access to home-based care, treatment adherence support, counselling, transport to health centres and nutritional support. While poverty is both a determinant and a frequent outcome of HIV and AIDS, these are essential care and support services that are essential to ensuring an effective and comprehensive response to the epidemic.

A National AIDS Spending Assessment for Zambia reported a 52% drop in funding available for CSOs for HIV between 2005 and 2006, a decline tentatively attributed to changes in the prevailing aid architecture and transitions in the resource environment [68]. This trend is being mirrored globally [69]. ‘Gold standards’ for sustainability are not appropriate as there may be wide variability in what can or should be sustained, depending on project type, setting or resources [24]. Therefore experience from Zambia can provide guidance but not a template for funding mechanisms to ensure sustainability. There is now a need to go beyond these dichotomies, so that interventions have a dual aim – to deliver services and to build on and strengthen community capacities.

To ‘sustain’ has been defined as ‘to supply with sustenance and to nourish’ [24]. While all CBOs delivering HIV support services in Mumbwa had existed prior to CRAIDS and did not appear to be wholly dependent on donor funds for their survival, CRAIDS nurtured them and enabled them to expand their deliver and community coverage of essential services for people living with HIV and AIDS at a modest cost, which others (the formal health and social sector services) were not in a position to deliver. It appears that many of the achievements under CRAIDS were not adequately captured and evaluated, after it ceased. An important message of this paper is to point to the importance of considering the effects of community-targeted interventions on the socio-cultural, political and economic environments, and on the resources and commitments of the actors within a community, which will determine the long-term sustainability of community actions in the health, HIV and social sectors [16]. In Mumbwa, this study shows that local community and district actors remained committed to delivering these care and support activities, two years after external funding ceased. It remains to be seen how long this commitment can be sustained without a similar and sustained commitment and financial support from the national and international actors that have the resources and ultimate responsibility to eliminate HIV and AIDS.

## Endnotes

^a^ We categorise CBOs as bottom-up forms of Civil Society Organisations (CSOs) that have emerged from the community, and usually deliver ‘soft’ services such as home-based care visits, and other prevention, care and support services for people living with HIV/AIDS and orphans and vulnerable children (OVCs). They are often less informal and less structured than nongovernment organisations (NGOs), which are a more formal form of CSO that sometimes lack the strong community roots of CBOs. Similarly, ‘community’ is a widely-used term that has no single or fixed definition. Communities are formed by people who are connected to each other in distinct and varied ways [13].

^b^ The CRAIDS Regional Facilitators’ primary role was assisting with field appraisal and M&E of CRAIDS projects in the districts.

^c^ DATFs are the structures responsible for the coordination, support and monitoring of HIV/AIDS services at the district level. Their role is also to facilitate training, resource mobilisation and advocacy. CRAIDS funded the position of the DACA, which has since been taken over by the National AIDS Council. DATFs existed prior to the ZANARA project.

## Abbreviations

ART: Antiretroviral treatment; CATF: Community AIDS Task Force; CBO: Community Based Organisation; CSO: Civil Society Organisation; CRAIDS: Community Response to AIDS; DACA: District AIDS Coordination Advisor; DATF: District AIDS Task Force; HBC: Home-based care; IGA: Income Generation Activity; MAP: World Bank Multi-Country AIDS Program; MDG: Millennium Development Goals; NAC: National AIDS Council; NARF: National AIDS Reporting Forms; NGO: Non-government organisation; OVC: Orphans and Vulnerable Children; PEPFAR: US Presidents’ Emergency Plan for AIDS Relief; ZANARA: Zambia National Response to HIV/AIDS; ZAMSIF: Zambia Social Investment Fund; ZNAN: Zambia National AIDS Network.

## Competing interests

The authors declare that they have no competing interests.

## Authors’ contributions

AW participated in study conception and design, data collection, data analysis and interpretation, and led on drafting the article. CM participated in study design, data collection, data analysis and interpretation, and drafting of the article. RB participated in study conception and design, data analysis and interpretation, and drafting of the article. JH participated in data collection, data analysis and interpretation, and drafting of the article. All authors have seen and approved the final version.

## References

[B1] Southern African AIDS Trust Strategic Framework 2008–2013 2008 Johannesburg

[B2] The AIDS Support Organisation (TASO) Living Positively with AIDS 1991 Uganda

[B3] Siamwiza R Analysis and Financing for the National HIV and AIDS Response: Civil Society Component 2007 Lusaka: A consultancy for the National HIV/AIDS/STI/TB Council, funded by DfID through the STARZ Programme

[B4] Treatment Advocacy and Literacy Campaign (TALC) Zambia From Fear to Hope. AIDS Care and Prevention at Chikankata Hospital 1992 Zambia

[B5] McIntyre K Carey S Assessing the Contribution of Civil Society Organisations to the National Response on HIV and AIDS in Zambia, 2006–2008 2009 Lusaka: NAC Zambia

[B6] World Health Organization Declaration of Alma-Ata 1978 USSR: International Conference on Primary Health Care, Alma-Ata 6 12

[B7] UNGASS Declaration of Commitment on HIV/AIDS 2001 United Nations General Assembly Twenty-sixth Special Session Doc: A/s – 26/L2, New York

[B8] World Health Organization The World Health Report 2008. Primary Health Care. Now More Than Ever 2008 Geneva

[B9] Lawn JE Rohde J Rifkin S Were M Paul VK Chopra M Alma-Ata 30 years on: revolutionary, relevant, and time to revitalise Lancet 2008 372 9642 917 927 10.1016/S0140-6736(08)61402-6 18790315

[B10] Gruen RL Elliott JH Nolan ML Lawton PD Parkhill A McLaren J Sustainability science: an integrated approach for health programme planning Lancet 2008 372 9649 1579 1589 10.1016/S0140-6736(08)61659-1 18984192

[B11] Torpey K Mwenda L Thompson C Wamuwi C Van Damme W From project aid to sustainable HIV services: a case study from Zambia J Int AIDS Soc 2010 13 16 19 10.1186/1758-2652-13-16 20529255 PMC2890667

[B12] Resch S Lee M Kombe G Sinyinza E Sustainability of HIV/AIDS Services in Zambia 2008 Bethesda: Health Systems 20/20 Project, Abt Associates Inc

[B13] Global Fund to Fight AIDS, TB and Malaria Community Systems Strengthening Framework 2010 Geneva

[B14] Low-Beer D Sempala M Social Capital and Effective HIV Prevention: Community responses Global Health Governance 2010 4 1

[B15] Harman S The World Bank: Failing the multi-country AIDS program. Failing HIV/AIDS Global Governance 2007 13 485 492

[B16] Morgan L Community participation in health: perpetual allure, persistent challenge Health Policy Plan 2001 16 3 221 230 10.1093/heapol/16.3.221 11527862

[B17] Rifkin S Lessons from community participation in health programmes Health Policy Plan 1986 1 3 240 249 10.1093/heapol/1.3.240

[B18] Perez D Lefevre P Romero MI Sánchez L De Vos P Van der Stufyt P Augmenting frameworks for appraising the practices of community-based health interventions Health Policy Plan 2009 24 5 335 341 10.1093/heapol/czp028 19549795

[B19] Putnam RD Bowling Alone: America’s declining social capital J Democr 1995 6 65 78

[B20] Social Capital Research http://www.socialcapitalresearch.com/definition.html

[B21] Brett EA Participation and Accountability in Development Management J Dev Stud 2003 40 2 1 29 10.1080/00220380412331293747

[B22] Brownill S Carpenter J Participation and Planning: Dichotomies, rationalities and strategies for power Town Planning Review 2009 78 4 401 428

[B23] Weiss H Coffman J Bohan-Baker M Evaluation’s Role in Supporting Initiative Sustainability Prepared for the fifth biannual meeting of the Urban Seminar Series on Children’s Health and Safety on the topic of “Strategies to Ensure the Continued Success of Large Scale Initiatives 2002 Harvard University, Cambridge, Massachusetts 5 6

[B24] Shediac-Rizkallah MC Bone LR Planning for the sustainability of community-based health programs: conceptual frameworks and future directions for research, practice and policy Health Educ Res 1998 13 1 87 108 10.1093/her/13.1.87 10178339

[B25] La Fond A Sustaining Primary Health Care 1995 London: The Save the Children Fund

[B26] Bossert TJ Can they get along without us? Sustainability of donor-supported health projects in Central America and Africa Soc Sci Med 1990 30 9 1015 1023 10.1016/0277-9536(90)90148-L 2336568

[B27] Sarriot EG Winch PJ Ryan LJ Bowie J Kouletio M Swedberg E LeBan K Edison J Welch R Pacqué MC A methodological approach and framework for sustainability assessment in NGO implemented primary health care programs Int J Health Plann Manage 2004 19 1 23 41 10.1002/hpm.744 15061288

[B28] Bracht N Kingsbury L Community organization principles in health promotion at the community level Health Promotion at the Community Level 1990 15 66 68 Sage sourcebooks for the human health services series. California: Sage

[B29] Flynn BS Measuring community leaders perceived ownership of health education programs: initial tests of reliability and validity Health Ed Research 1995 10 27 36 10.1093/her/10.1.27 10150420

[B30] Ooms G Stuckler D Basu S McKee M Financing the Millennium Development Goals for health and beyond: sustaining the ‘Big Push’ Glob Heal 2010 6 6 17 10.1186/1744-8603-6-6 PMC2958957 20932274

[B31] National HIV/AIDS Counci Zambia Country Report. Monitoring the Declaration of Commitments on HIV and AIDS and the Universal Access. Biennial Report. Submission to the United Nations General Assembly Special Session on HIV and AIDS 2012 Lusaka

[B32] United Nations Development Programme Zambia Human Development Report 2011. Service Delivery for Sustainable Human Development 2011 Lusaka: Zambia Ministry for Finance and National Planning

[B33] Global Fund to Fight AIDS, Tuberculosis and Malaria, Macro International Global Fund Five Year Evaluation, Study Area 3. The Impact of Collective Efforts on the Reduction of the Disease Burden of AIDS, Tuberculosis and Malaria 2009 Geneva: Global Fund

[B34] World Health Organization Macroeconomics and Health: Investing in Health for Economic Development Report of the Commission on Macroeconomics and Health 2001 Geneva: WHO

[B35] Goldsbrough D Cheelo C IMF Programs and Health Spending: Case Study of Zambia Working Group on IMF Programs and Health Expenditures Background Paper 2007 Washington: Center for Global Development

[B36] The World Bank The World Bank Completion and Results Report – Zambia 2009

[B37] The World Bank The World Bank’s Commitment to HIV/AIDS in Africa. Our Agenda for Action, 2007–2011 2008 Washington: The World Bank

[B38] Edwards JC Feldman PH Sangl J Polakoff D Stern G Casey D Sustainability of partnership projects: a conceptual framework and checklist Jt Comm J Qual Patient Saf 2007 33 12 37 47 18277638 10.1016/s1553-7250(07)33122-x

[B39] Beery WL Senter S Cheadle A Greenwald HP Pearson D Brosseau R Evaluating legacy of community health initiatives: A conceptual framework and example from the California Wellness Foundation’s Health Improvement Initiative American J Eval 2005 26 150 165 10.1177/1098214005275627

[B40] Mancini JA Marek LI Sustaining community-based programs for families: conceptualisation and measurement Fam Relat 2004 53 4 339 347 10.1111/j.0197-6664.2004.00040.x

[B41] Eng E Parker E Measuring community competence in the Mississippi Delta: the interface between program evaluation and empowerment Health Educ Q 1994 21 2 199 220 10.1177/109019819402100206 8021148

[B42] Yin RK Case study research: Design and methods 2003 3 California: Sage

[B43] Gerring J What Is a Case Study and What Is It Good For? Am Pol Sci Review 2004 98 2 341 354

[B44] Miles MB Huberman AM Qualitative data analysis: A sourcebook of new methods 1994 2 California: Sage

[B45] Central Statistical Office Zambia 2010 Census of Population and Housing. Preliminary Population Figures 2011 Lusaka

[B46] Zambia National Response to HIV/AIDS (ZANARA) Project Community Response to AIDS Component. Project Implementation Manual 2005 Lusaka: Ministry of Finance and National Planning

[B47] Green LW Is institutionalisation the proper goal of grant-making? American Jnl of Health Promotion 1989 3 4 44 10.4278/0890-1171-3.4.44 22206394

[B48] Doyle C Patel P Civil society organisations and global health initiatives: problems of legitimacy Soc Sci Med 2008 66 9 1928 1938 10.1016/j.socscimed.2007.12.029 18291566

[B49] Harman S Bottlenecks and benevolence: how the World Bank is helping communities to ‘cope’ with HIV/AIDS Jnl Health Manag 2009 11 2 279 313 10.1177/097206340901100202

[B50] Briggs CJ Garner P Strategies for integrating primary health services in middle-and-lower-income countries at the point of delivery Cochrane Database Syst Rev 2006 19 2 CD003318 16625576 10.1002/14651858.CD003318.pub2

[B51] Msuya J Horizontal and vertical delivery of health services: what are the tradeoffs? 2005 Washington: World Bank

[B52] Atun RA Bennett S Duran A When do vertical (stand alone) programmes have a place in health systems? 2008 Copenhagan: World Health Organization on behalf of the European Observatory on Health Systems and Policies

[B53] Banerji D Political context of the work of international agencies – a fundamental shift in the approach to international health by WHO, UNICEF, and the World Bank: instances of the practice of “intellectual fascism” and totalitarianism in some Asian countries Int Jnl Health Services 1999 29 227 259 10.2190/RAB4-D873-99AM-ACJR 10379453

[B54] Stefani A Ruck N Managing externally assisted health projects for sustainability in developing countries Int Jnl Health Plan Manag 1992 7 199 210 10.1002/hpm.4740070305

[B55] Oliviera-Cruz V Kurowski C Mills A Delivery of health interventions: searching for synergies within the vertical versus the horizontal debate Jnl Int Dev 2003 15 67 86 10.1002/jid.966

[B56] Cairncross S Peries H Cutts F Vertical health programmes Lancet 1997 349 3 20 28988118

[B57] Goodman RM Steckler A A model for the institutionalisation of health promotion programs Fam Community Health 1989 11 63 78 10.1097/00003727-198902000-00009

[B58] Lehman U Sanders D Community health workers: What do we know about them? The state of the evidence on programmes, activities, costs and impact on health outcomes of using community health workers 2007 Geneva: World Health Organization

[B59] Community Health Worker Strategy in Zambia http://www.who.int/workforcealliance/forum/2011/29_Zambia.pdf

[B60] Zambia National Response to HIV/AIDS Project CRAIDS Annual Report 2006 Lusaka: Ministry of Finance and National Planning

[B61] Sanjana P Torpey K Schwarzwalder A Simumba C Kasonde P Nyirenda L Kapanda P Kakungu-Simpungwe M Kabaso M Thompson K Task-shifting HIV counselling and testing services in Zambia: the role of lay counsellors Hum Resour Heal 2009 7 44 10.1186/1478-4491-7-44 PMC2692981 19480710

[B62] Gorgens-Albino M Mohammad N Blankhart D Odutolu O The Africa Multi-country AIDS Program 2000–2006: Results of the World Bank’s Response to a Development Crisis 2007 Washington: The Global AIDS Monitoring and Evaluation Team of the Global HIV/AIDS Program; The World Bank

[B63] National HIV/AIDS Council National HIV/AIDS Strategic Framework 2011–2015 2010 Lusaka: Government of Zambia

[B64] Catterson J Claes L The Sustainability Enigma. Aid dependency and the phasing out of projects. The case of Swedish aid to Tanzania 1999 Stockholm: Management Perspectives International & Norstedts Trycker

[B65] Ugalde A Ideological dimensions of community participation in Latin American health programs Soc Sci Med 2002 21 1 41 53 4035407 10.1016/0277-9536(85)90286-2

[B66] Scanteam Support Models for Civil Society Organisations in Zambia 2007 Oslo

[B67] Lagarde M Haines A Palmer N The impact of conditional cash transfers on health outcomes and use of health services in low and middle income countries Cochrane Database Syst Rev 2009 7 4 CD008137 19821444 10.1002/14651858.CD008137 PMC7177213

[B68] National HIV/AIDS Council Zambia National AIDS Spending Assessment for 2005 and 2006. Final Draft Technical Report 2008 Lusaka: Ministry of Health

[B69] Larson HJ Bertozzi S Piot P Redesigning the AIDS response for long term impact Bulletin WHO 2011 89 846 852 10.2471/BLT.11.087114 PMC3209724 22084531

